# Proof of Concept of an Online EMG-Based Decoding of Hand Postures and Individual Digit Forces for Prosthetic Hand Control

**DOI:** 10.3389/fneur.2017.00007

**Published:** 2017-02-01

**Authors:** Alycia Gailey, Panagiotis Artemiadis, Marco Santello

**Affiliations:** ^1^School of Biological and Health Systems Engineering, Tempe, AZ, USA; ^2^School for Engineering of Matter, Transport, and Energy, Arizona State University, Tempe, AZ, USA

**Keywords:** myoelectric hand, neuroprosthesis, machine learning applied to neuroscience, neurorobotics, brain–machine interface

## Abstract

**Introduction:**

Options currently available to individuals with upper limb loss range from prosthetic hands that can perform many movements, but require more cognitive effort to control, to simpler terminal devices with limited functional abilities. We attempted to address this issue by designing a myoelectric control system to modulate prosthetic hand posture and digit force distribution.

**Methods:**

We recorded surface electromyographic (EMG) signals from five forearm muscles in eight able-bodied subjects while they modulated hand posture and the flexion force distribution of individual fingers. We used a support vector machine (SVM) and a random forest regression (RFR) to map EMG signal features to hand posture and individual digit forces, respectively. After training, subjects performed grasping tasks and hand gestures while a computer program computed and displayed online feedback of all digit forces, in which digits were flexed, and the magnitude of contact forces. We also used a commercially available prosthetic hand, the i-Limb (Touch Bionics), to provide a practical demonstration of the proposed approach’s ability to control hand posture and finger forces.

**Results:**

Subjects could control hand pose and force distribution across the fingers during online testing. Decoding success rates ranged from 60% (index finger pointing) to 83–99% for 2-digit grasp and resting state, respectively. Subjects could also modulate finger force distribution.

**Discussion:**

This work provides a proof of concept for the application of SVM and RFR for online control of hand posture and finger force distribution, respectively. Our approach has potential applications for enabling in-hand manipulation with a prosthetic hand.

## Introduction

A significant challenge faced in modern medicine is in replacing a lost hand for upper limb amputees. The human hand performs many complex functions in the activities of daily living. One area of challenge is in the tradeoff between functionality and ease of use. A prosthetic hand that performs more functions will generally require more cognitive effort from the user. In contrast, a prosthetic hand that is simpler to control generally has more limited functionality.

According to an epidemiology study by Dillingham and colleagues (1), from 1988 to 1996 about 134,000 Americans underwent upper limb amputations from trauma and another 29,400 Americans lost their upper limbs to dysvascular disease. Though there are already a number of different upper limb prostheses available, the rate at which individuals with upper limb loss stop using the prostheses is significant. A meta-analysis by Biddiss and Chau (2) showed abandonment rates of 26% in body-powered prosthesis users and 23% in electrically powered prosthesis users. Major reasons for these abandonment rates include heavy weight, lack of functionality and durability, discomfort, poor cosmetic appearance, and finally too much effort required to control the prosthesis.

One of the main remaining challenges for prosthetic hand developers is in allowing the user to reliably control many different hand movements without too much cognitive effort. Body-powered systems are reliable, but their harness system can result in fatigue and strain (2). Furthermore, body-powered prostheses are limited in their functionality. Control systems based on electroencephalographic (EEG) signals can be used to control prosthetic hands for above-elbow amputees and paralyzed individuals (3, 4). However, the implementation of these systems tends to be challenging because EEG signals are associated with many other behaviors besides hand motion, such as proximal musculature involved in hand transport, trunk movement, and so forth. Other methods are being developed to extract signals from within the brain or peripheral nerve tissue, but such methods are invasive and expensive (5).

Myoelectric systems are based on electromyographic (EMG) activity of residual muscles following an amputation and offer several advantages relative to the above-described systems. Specifically, EMG-controlled systems are non-invasive, and they take advantage of signals recorded from residual muscle activity that is specifically involved in the task. Numerous systems have been developed for recording surface EMG signals from the upper limb and extracting features to predict in real-time grasp postures and/or forces, or for individuals with upper limb loss, predicting the user’s intended hand movement. Castellini et al. (6) demonstrated the use of EMG in predicting hand postures in healthy able-bodied individuals. Castellini et al. (7) further demonstrated that EMG signals from the residual muscles of amputees can predict five different imagined grasp poses to accuracies around 79–95% and grip force with accuracies between 7 and 17% normalized root mean square error (NRMSE) (7). The grasp poses included open hand, closed fist, 2-digit pinch, tripod (3-digit) grip, and index finger pointing.

In the same year, Yang et al. (8) demonstrated the use of EMG in the forearm to predict one of 18 possible combinations of finger flexion/extension for the thumb, index, and middle finger. It was found that while the classification accuracy was quite high when training and testing on data collected within the same session, training on data in an earlier data collection session and testing on a later session yielded much lower classification accuracies (50–60%). Subsequently, Castellini and Kõiva (9) demonstrated the use of a myoelectric control system that allowed 12 able-bodied subjects to modulate individual finger forces when the hand lay flat on a surface with each finger placed on top of force sensors. Although this system is a good proof of principle of myoelectric control of finger forces, this type of control was not demonstrated in a grasping task when the subject’s hand is not laid flat on a surface, but rather is in a fist. In addition, this work does not account for wrist rotation during grasping tasks, although future work should focus on testing the robustness of our decoder across wrist postures that are commonly found in activities of daily living. Later work by Cipriani et al. (10) examined real-time myoelectric control of grasp types by individuals with upper limb loss with nine EMG electrodes placed along either side of the residual forearm muscles. Predicted gestures included open hand, closed fist, thumbs up, index finger pointing with extended thumb, flexed thumb with four extended fingers, and 3-digit grip. Average online control classification accuracy was 79% for transradial amputees and 89% for able-bodied subjects. However, this work did not examine online control of digit forces.

The present work attempts to expand upon the work of Castellini et al. (7) by implementing online control of hand postures—as opposed to offline cross-validation—and online control of individual finger forces, which allow subjects to modulate the distribution of force across the fingers of the prosthetic hand. The system is programed in Matlab, and therefore does not implement true real-time control. However, the delay between a change in EMG signals and a change in desired hand motion is only 0.3 s. For a proof-of-concept demonstration of our approach, we used the commercially available i-limb hand (Touch Bionics) because it has a separate motor for each digit and each digit can exert seven different levels of grip forces, thus allowing for some degree of force modulation by individual digits. We examined several grasping tasks, including lifting the object using a chosen grasp type (2-digit, 3-digit, or whole-hand grasp), alternating between grasp types while holding the object, and modulating the grip force during the object holding phase. Because the i-limb hand has certain limitations (limited speed of motion, limited ability to exert a discrete set of forces per digit), we tested this system mostly by using a computer program to give online feedback of finger forces and grasp type for each loop iteration, while simultaneously instructing the subject to perform different hand poses. The system’s performance is demonstrated on able-bodied individuals before future testing on individuals with upper limb loss.

## Materials and Methods

### Subjects

Eight right-handed subjects (age: 23.5 years, SD: ±3.42, five males, three females) participated in the study. We recruited subjects who identified themselves as right handed. Subjects had no history or record of neurological disorders and had never performed tasks involving myoelectric control of an external device. Subjects gave informed written consent to participate in the experiments. The experiments were approved by the Institutional Review Board at Arizona State University and were in accordance with the Declaration of Helsinki. Each experimental session (one session/subject) lasted approximately 1.5–2 h.

### Experimental Protocols

**Figure 1:**
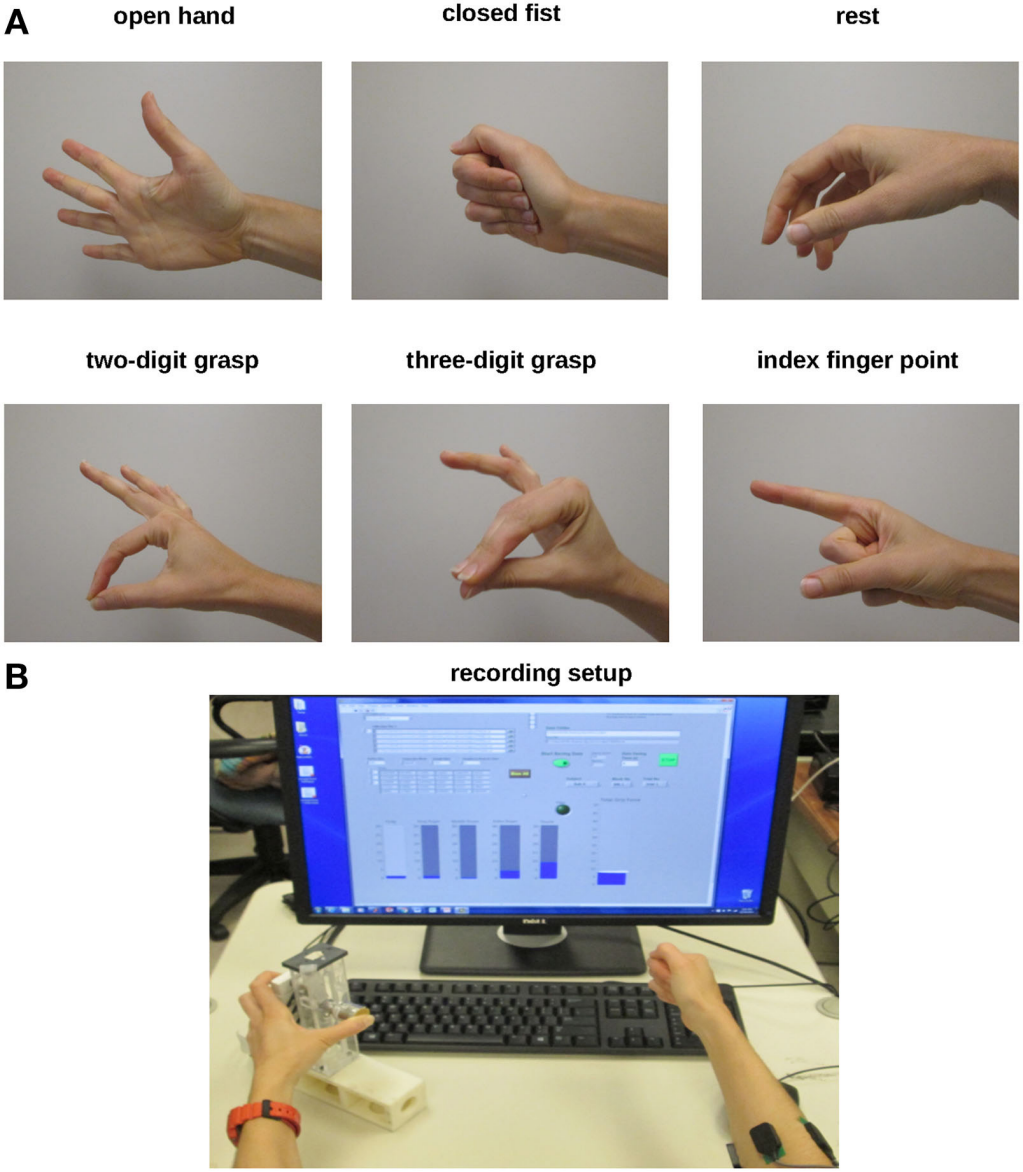
**Experimental protocols**. **(A)** For Experiment 1 (Hand postures), subjects were asked to shape their right hand to create six postures (open hand, closed fist, rest, 2-digit grasp, 3-digit grasp, and pointing). **(B)** For Experiment 2 (Digit forces), subjects were asked to change the distribution of finger forces of their right hand while grasping a sensorized object with the left hand. A computer monitor was used to display force data recorded from the sensorized grip device grasped with the left hand. Subjects were given force feedback for each digit in the form of rising bars (one/digit, bottom left display) as well as for all digits (total grip force, bottom right display). For both experiments, we recorded electromyographic activity through surface electrodes placed on the right forearm.

We asked subjects to perform two sets of tasks. In the first set of tasks, subjects were asked to perform a series of grasping and finger pointing tasks (*Task 1: hand postures*; Figure 1A). In the second set of tasks, subjects were asked to vary the distribution of normal forces among the fingers in a closed fist (*Task 2: digit forces*). For both tasks, we recorded EMG signals from five surface EMG electrodes and extracted features from these signals to train a one-against-one support vector machine (SVM). This SVM was used to distinguish hand poses, and a random forest regression (RFR) was used to predict each of the five digit forces. Subjects performed both tasks in the same experimental session. Below we describe the procedures for both training and testing of our EMG decoder system.

### EMG Recording, Signal Processing, and Feature Extraction

We placed five surface EMG electrodes (Delsys) around the circumference of the forearm just below the elbow (Figure 1B). The electrodes were roughly equally spaced apart. At least one EMG electrode was placed over the m. extensor digitorum superficialis (finger extensor) on the dorsal surface of the forearm. The other electrodes did not target specific muscles. A reference EMG electrode was placed directly over the lateral epycondyle of the humerus. Prior to EMG electrode placement, the area was cleaned with rubbing alcohol pads. We did not target specific muscles because this approach is not always feasible when using surface EMG from residual muscles in individuals with upper limb loss. Specifically, availability of specific target muscle depends on the extent and state of residual muscle fibers following amputation which, in turn, may affect the EMG signal quality and the extent to which it can be used for hand posture or grasp force decoding. Thus, we aimed at using a muscle-independent EMG decoding approach to resemble a more realistic scenario of extracting features from non-specific forearm muscles.

Our preliminary online and offline testing showed that increasing the number of electrodes did not improve the hand pose classification accuracy. We had also performed preliminary offline testing on a high-resolution electrode array (90 channels) and found that hand pose classification was more difficult due to lower signal quality. Furthermore, work by Castellini et al. (7) showed that five electrodes placed around the circumference of the forearm allowed for prediction of desired hand pose in three amputees. This work, as well as our study, suggested that the quality of the EMG signal might be more important for hand pose and finger force decoding than the number of EMG channels.

All EMG signals during training and testing were analyzed in individual 50-ms non-overlapping time windows for the purpose of enabling online control. Although time windows longer than 50 ms may improve prediction accuracy, we chose a 50-ms time window length based on the need to reduce control delays due to data processing (see below). However, we note that pilot testing revealed that longer time windows would not have significantly improved prediction accuracy. Furthermore, Castellini et al. (7) reported that a time window of 50 ms was sufficiently long to make predictions of desired hand motions in amputees. Control was quasi real time as the processing delay was about 0.3 s. For each EMG signal-recording interval, the computed signal average was subtracted to center the signal amplitude about 0. Next, all points in the time interval were normalized to a range spanning −1 to +1 where a value of ±1 represents the EMG magnitude recorded during maximum voluntary contraction (MVC). MVC EMG was recorded by asking subjects to perform six maximum isometric force contractions: isometric finger flexion, finger extension, wrist flexion, wrist extension, wrist abduction, and wrist adduction. For each EMG channel, the root mean square (RMS) was computed for each 50-ms time window throughout the entire MVC trial. The maximum RMS value computed for each channel during the MVC recording was used as the EMG magnitude representative of MVC for that channel. All signals collected in that channel in subsequent recordings were centered at 0 and then divided by the MVC magnitude.

Electromyographic signals were amplified in hardware (gain: 1,000; Delsys Bagnoli-8 EMG System) before being digitized and analyzed in software. After subtraction of the mean and normalization of the EMG signal, 60 Hz line noise was filtered by passing the signal through a 60-Hz notch filter with 3-dB cutoff and bandwidth set to 20 Hz. Next, features were extracted from each filtered 50-ms time window. We explored various EMG signal features presented in previous work [e.g., Zecca et al. (11) and Khushaba et al. (12)] including mean absolute value, variance, Willison amplitudes, mean of amplitudes, auto-regressive coefficients, and other novel features presented by Khushaba et al. (12). Of these features, three were found to be most informative for predicting hand posture. The first chosen feature is the RMS, computed according to Eq. 1:

(1) $$\text{RMS}=\sqrt{\frac{1}{N}\sum_{t=t_{1}}^{t_{2}}x^{2}(t)}$$

where the term *x*(*t*) denotes the EMG signal value at time *t*.

The next two features were derived by Khushaba et al. (12). These features were computed by the zero-order moment (same as RMS above), *m*_0_, the second-order moment, *m*_2_, and the fourth-order moment, *m*_4_. These moments were computed according to Eqs 2–4:

(2) $$m_{0}=\int_{t-T}^{T}x^{2}(t)\mathrm{dt}$$

(3) $$m_{2}=\int_{t-T}^{T}{\left(\frac{\mathrm{dx}}{\mathrm{dt}}\right)}^{2}\;\mathrm{dt}$$

(4) $$m_{4}=\int_{t-T}^{T}{\left(\frac{d^{2}x}{dt^{2}}\right)}^{2}\;\mathrm{dt}$$

The variable *T* is the length of time for one time window. After computing the moments, the irregularity factor, IF, and the wavelength variable, WL, were computed according to Eqs 5 and 6:

(5) $$IF=\sqrt{\frac{m_{2}^{2}}{m_{0}m_{4}}}$$

(6) $$\text{WL}=\int_{t-T}^{T}\left|\frac{\mathrm{dx}}{\mathrm{dt}}\right|\mathrm{dt}$$

One of the features (*f*) used was dependent on the zero-order moment or RMS, computed according to Eq. 7:

(7) f2=log(m0N)

The variable, *N*, is the number of samples within each time window. The third feature type was computed according to Eq. 8:

(8) f3=log(IFWL)

Each type of feature was computed not only for each individual EMG channel but also for each combination of channel pairs where the feature value of one channel would be subtracted from the feature value of the other channel, thus yielding 15 features of each feature type. To identify non-redundant features, we performed linear correlation between feature pairs. As many features were highly correlated with each other, we did not use all features for hand posture and digit force prediction. Therefore, we chose the 15 features characterized by the weakest correlation with each other. For each pair of features that were highly correlated, we chose the feature that changed the most between grasp types. We found that the features that changed the most between grasp types also were highly correlated with each other, and were therefore redundant. Selecting features least correlated with each other yielded less redundancy, and therefore more information. As such, the same set of features was used in distinguishing all pairs of hand poses.

### Force Data Recording and Processing

Normal forces exerted by each digit were measured by five force/torque (F/T) sensors (ATI Industrial Automation) mounted on a grip device grasped by the left hand. Four F/T sensors (Nano-17) were mounted on the finger side of the grip device, and an F/T sensor (Nano-25) was mounted on the thumb side. During recording sessions, the subject grasped the force-sensing device with one digit on each force sensor, and force data were collected synchronously with EMG data to form a mapping between EMG and each digit force.

### EMG and Force Data Processing

Electromyographic and force data were acquired at 1 kHz by 12-bit analog-to-digital converter boards (NIDAQ PCI-6225, National Instruments; sampling frequency: 1 kHz). EMG data recording was performed through Matlab (Mathworks), which forces data recording through LabVIEW (version 8.0, National Instruments). EMG data and force data were synchronized by a trigger pulse sent by the LabVIEW program to the NIDAQ board at the start of the recording. This pulse appeared in the EMG recording in Matlab.

### Training Data Collection

After recording MVC EMG, the subject was prompted to relax the hand for 1 min while EMG signals were recorded. RMS was computed for each 50-ms time window of the resting period and the resting threshold was computed as 1.5 times the average magnitude of the EMG signal in each channel. When the RMS of all five EMG channels was below their resting threshold, the computer program predicted that the hand was at rest.

The EMG decoder predicts one of six possible hand poses. The hand poses are shown in Figure 1A. We chose these poses to capture basic grasp types [2- and 3-digit precision grasps where the thumb contacts the fingertips; closed fist to approximate a “power” grasp (13)], as well as non-prehensile hand postures (rest, open hand, and index finger pointing). To form a mapping between EMG signals and hand pose/digit forces, data were collected from 12 training trials lasting 30 s each. For trials 1 and 2, the subject extended all fingers. About 15 s after the start of the recording, the subject was told to co-contract the hand muscles while keeping fingers extended for about 7–8 s. For trials 3 and 4, the subject shaped the hand into a 2-digit grasping pose where the thumb and index finger pressed against each other at the tips and the other three fingers were extended (Figure 1A). Fifteen seconds after the start of the recording, the subject was told to increase the pinch force for about 7–8 s. For trials 5 and 6, the subject did an index finger pointing (Figure 1A). Subsequently, the subject was told to increase and then decrease the muscle co-contraction. For trials 7 and 8, the subject did a 3-digit grasp (index finger and middle finger pressing against the tip of the thumb while ring finger and little finger were extended; Figure 1A). Fifteen seconds after the start of the recording, the subject was told to increase the pinch force.

For trials 9–12, subjects made a fist with the ipsilateral hand and grasped the force-sensing object with the contralateral hand (Figure 1B). The subject was asked to keep flexion forces approximately the same for both hands. For trial 9, the subject began by making a fist using a small grip force, and then the subject shifted most of the force onto the index finger with the ipsilateral hand while still maintaining a closed fist. With the contralateral hand, the subject exerted most or all of the pressure on the force sensors with the thumb and index finger to teach the RFR that most of the force was concentrated on the index finger and thumb. The same procedure was repeated for the middle finger. For trial 10, the process was repeated for the ring finger and little finger. For trials 11 and 12, the subject started the trial with the ipsilateral hand in a relaxed fist and the contralateral hand exerting minimal force on all five force sensors. The subject was then prompted to ramp up the flexion force across all digits on both hands to a moderately high value, maintain the higher grip force for about 5 s, and then ramp the force back down.

The rationale for having subjects make a fist with the right ipsilateral hand during training while grasping the force sensors with the contralateral hand was that this approach is more feasible for training individuals with unilateral (ipsilateral) upper limb loss. Specifically, these individuals can use the intact (contralateral) hand to grasp force-sensing object while sending similar motor commands to the ipsilateral hand to trying to match forces across the two limbs.

One of the goals of the system in the current study is to allow the user to grasp the object with the prosthetic hand by simply making a fist with the real hand, and then pressing the fingers into the palm in order to modulate the distribution of force across the fingers. EMG signals can change depending on the size of the object being grasped. Therefore, if EMG signals are recorded while the subject is grasping a force-sensing object of a certain size, then the subject may need to grasp an object of the same size during online control in order to generate the desired finger forces.

### Training the Machine Learning Classifiers and Regressions

**Figure 2:**
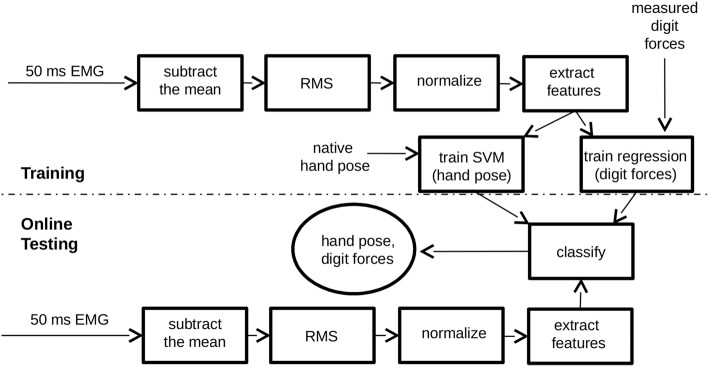
**Electromyographic (EMG) decoding system block diagram**. For training, the system takes three inputs: EMG signal, force measurements from each digit of the hand, and hand pose. For testing, the system uses only one input, EMG, to predict hand pose and each digit force in real time. Each rectangle in the diagram represents a data processing step. The circle represents the system output. Above the dashed line is the training portion of the system, which forms a mapping between EMG and hand pose as well as a mapping between EMG and each of the five digit forces. Below the dashed line is the portion of the system that inputs EMG, extracts features, and generates a prediction of hand posture and digit forces for online control.

Figure 2 shows a block diagram of the training portion and testing portion of our system. 15 The training portion inputs data from the subject to form a mapping between EMG signal and 16 hand pose, and a mapping between EMG signal and finger forces. The testing portion of the 17 system inputs EMG signals and makes online predictions of hand pose and finger forces.

To distinguish among the five hand postures, we used a one-against-one radial basis kernel SVM. Such a system consists of one SVM binary classifier to distinguish each pair of hand poses. When one hand pose is guessed in favor of the other one, the hand pose receives a vote. The hand pose receiving the most votes across the different SVM classifiers gets selected. The hyperparameters were determined by offline testing on preliminary pilot data. Such testing would train on some data and test on the other data. The classification errors were computed for each set of hyperparameters, and the hyperparameters yielding the lowest classification errors were selected. For more information on SVMs and the one-against-one multiclass classification methods, the reader is referred to Hsu and Lin (14). To distinguish each pair of poses, 15 features were selected with the lowest correlation with each other. For digit force predictions, the 15 RMS features were used to train a RFR, which used 50 trees and all 15 features/tree. For a description of RFR algorithm, see Breiman (15). A separate RFR was trained for each digit force.

### Online Myoelectric Control: Finger Force Prediction through a Computer Program

Regulation of force is difficult with the i-limb hand because (1) the i-limb hand commands only consist of integer values from −7 to +7, thereby limiting the resolution of force exertion and (2) if force is too high, it is difficult to reduce the force on a given finger by a specific amount unless extensive calibration is employed to determine the duration of the extension command necessary to decrease the force by a specific amount without losing contact. Furthermore, (3) the wireless Bluetooth-based communication required to receive commands tended to be unstable in our setup. Therefore, most of the online testing of the quality of myoelectric control was performed using a custom-made computer program. This program printed to the screen the predicted flexion forces for each digit during each loop iteration (~3 loop iterations/second). The computer program prompted the subject either to perform grasping tasks or to make a fist and vary the force distribution among the fingers.

For simulated grasping tasks using the computer program, 50 ms of EMG data were recorded for each loop/iteration. EMG signal features were extracted and input into the SVM classifiers for hand posture prediction, and RFRs for digit force prediction. Flexion and extension forces for each digit were printed to the screen at the end of the loop iteration. A force value greater or lower than 0 indicated predicted flexion or extension, respectively. A value equal to 0 indicated a predicted resting state. If all printed digit forces were negative, then the predicted hand pose was open hand. If all printed digit forces were positive, then the predicted hand pose was closed fist. If only the top 2-digit forces (index finger and thumb) were positive, then the predicted hand pose was a 2-digit pinch grip. If only the index finger force was negative and all other forces were positive, then the predicted hand pose was an index finger point (which could be performed while grasping an object).

Initially, the subject was given time to familiarize with the computer program used to display predicted finger forces by learning to associate the printed forces on the screen with specific hand poses. If digit force control was not sufficient, then training would be repeated for specific hand poses that were found to be more difficult to predict. To test the quality of myoelectric control, commands were posted on the screen to the subject about what hand posture to adopt and what target force to reach during grasping.

The computer program would summate the normal forces of the digits in contact and print the total grip force in the space below the individual digit forces. When the subject was asked to grasp, a target grasping force was printed to the screen in the area below the predicted total grip force. We chose submaximal target grasping forces (ranging from 6 to 30 N for a 2-digit and power grasp, respectively) as these are typically associated with activities of daily living. An example of force feedback (printed to the screen once per loop iteration) is shown below:

INDEX-THUMB GRASP
Thumb: 5.62
Index finger: 4.32
Middle finger: 0.05
Ring finger: −1
Little finger: −1
9.944 N
Target Force: 6 N

Each grasp task consisted of 100 iterations, at roughly 300 ms/loop iteration. Below is the list of commands given to the subject in chronological order:

Relax, Open Hand, Index-thumb grasp (target force = 6 N, 12 N), Open Hand, Relax
Open Hand, Three-digit grasp (target force = 10 N, 20 N), Open Hand, Relax
Open Hand, Power grasp (target force = 30 N, 25 N, 20 N, 15 N, 10 N), Open Hand
Relax, Open Hand, Power grasp (target force = 12 N), Index finger point (target force = 12 N), Power grasp, Index finger point, Power grasp, Open hand, Relax
Open Hand, Power grasp, Index-thumb grasp, Power grasp, Index-thumb grasp, Power grasp, Open Hand, Relax

To factor out response time of subjects to the change in command, the program paused for 4 s whenever there was a change in the command given to the subject.

For the second set of exercises, subjects made a fist the entire time and then varied the magnitude and distribution of flexion force across the digits. For the first 20% of iterations, subjects began with a relaxed fist (minimal flexion forces) and when prompted, made a tight fist. Every 20 iterations, subjects transitioned from tight to relaxed fist, or from relaxed to tight fist. For the second 20% of iterations, subjects were prompted to shift the force to the little finger. For the third 20% of iterations, subjects were prompted to shift the force to the ring finger. For the fourth 20% of iterations, subjects shifted the force to the middle finger. For the fifth 20% of iterations, subjects shifted the force to the index finger. Both sets of trials were performed five times. Subjects alternated between grasping task rounds and force-shifting task rounds, making a total of 10 rounds. We accepted all attempts at achieving the target force. The measure of how close the subject attained the target force was captured the NRMSE, which was computed as the square root of the difference between the target and predicted force divided by the target force.

### Testing with the i-Limb Hand

For practical demonstration purposes, the myoelectric control system was tested on a commercially available prosthetic hand, the i-limb (Touch Bionics). For each loop iteration, commands were wirelessly sent to the i-limb hand where the integer value of the flexion command was proportional to the predicted flexion force. The subject was asked to grasp the 5-digit force-sensing object (one force sensor/digit) using a 5-digit grasp, 2-digit pinch grip, and a 3-digit tripod grip. The subject was only required to complete one successful trial for each grasp type. Finally, the subject was prompted to alternate grasp types during object hold. For this trial, the subject used myoelectric control to grasp the object with five digits. Upon command, the subject released the index finger to do an index finger point, and then upon command, the subject returned to the 5-digit grasp. Next, the subject was prompted to release 2 or 3 of the fingers and transition to a 3-digit or 2-digit grasp without losing contact. Finally, the subject released the object. For all grasping tasks with the i-limb hand, a successful trial was defined as a trial during which the subject could hold a grasp for about 5 s without unwanted hand opening, and then release the grasp on verbal command.

To quantify the level of difficulty in completing simple grasping tasks with the i-limb hand, subjects were asked to rate each i-limb task level of difficulty on a scale from 1 to 5. On the scale, 1 = “easy,” 2 = “able to do the task,” 3 = “able to do the task, but with effort,” 4 = “moderately hard,” and 5 = “very hard or could not do.”

### Data Analysis: Predicted Finger Forces and Force Distributions

For trials concerned with grasping tasks, variables of interest included the NRMSE of the grip force across grasp types, and confusion matrix that relates predicted hand posture to desired hand posture. The error of the grip force was computed as the difference between the total predicted grip force and the target force. Total grip force was computed as the summation of normal forces across all digits that were supposed to be flexed. For hand pose predictions, the overall percent accuracy was computed as the percent of the loop iterations during which the predicted hand pose was correct out of the total of 500 loop iterations. Another variable of interest is the number of times that an open hand is predicted when the correct hand pose is a grasp. Such a situation would indicate unwanted loss of contact.

For trials concerned with force distribution across the fingers, the finger forces were summated for each loop/iteration, and then the fraction of the total finger force contributed by each finger was computed. For loop iterations 101–200, the little finger was expected to contribute the highest fraction of total finger force out of all the other fingers. Similarly, for loop iterations 201–300, the ring finger was expected to contribute the highest fraction of total finger force. For loop iterations 301–400, the middle finger was expected to contribute the highest fraction of total finger force. For loop iterations 401–500, the index finger was expected to contribute the highest fraction of total finger force. Data analyses examined the extent to which the force distribution across fingers changed as the subject was prompted to shift the flexion force from one finger to the next. Force distributions were measured as the percent of total finger force that was contributed by each finger during each time epoch. Force distribution index was used as an additional measure of force distribution, with a value of −1 for all force concentrated on the little finger, a value of +1 for all force concentrated on the index finger and a value of 0 for evenly distributed forces.

When force distribution changed across fingers, the myoelectric control system may predict an incorrect grasp type. To investigate this issue, the variable of interest was the percentage of the time that the 5-digit (correct) hand posture was predicted.

### Statistical Analysis

The first statistical analysis focused on performance during the in-hand force-shifting task. A repeated-measures analysis of variance (ANOVA) was performed on the force distribution index (range: −1 to 1) with three within-subjects factors: round of trials (*Round*; five levels), time epoch within each round (*Time epoch*; five levels), and first half versus second half of each time epoch (*Sample*; 2 levels). Subjects performed five rounds of the force-shifting tasks. Changes in force distribution across rounds indicate whether there was significant change in performance with practice. Changes in force distribution across time epochs indicate whether there was a significant change in force distribution across the fingers when subjects were prompted to change their force distribution from one finger to the next. The purpose of analyzing the factor *Sample* was to examine if performance was dependent on the length of time that subjects were asked to maintain a force distribution concentrated on one particular finger. Because each loop/iteration lasted approximately 300 ms, each time epoch was about 30 s long.

A second ANOVA was performed on overall hand pose prediction accuracy for the grasping tasks using one within-subject factor (*Round*; five levels). Results of this analysis would indicate whether overall prediction accuracy improved with practice.

## Results

The EMG control system design presented here is for demonstrating the use of machine learning techniques to decode five surface EMG signals from the forearm to predict desired hand motion. A machine learning-based mapping was created between EMG signal features and individual finger movements, allowing online control of individual finger movements in a robot hand. The end goal is to allow subjects to open the hand, grasp an object using a chosen grasp type, and execute an index finger pointing for gesturing or typing. When grasping, subjects were told to use the EMG decoder system to modulate not only the amount of grip force but also the distribution of grip force across the fingers. To test system performance, subjects were prompted to perform grasping tasks and finger pointing tasks with a virtual hand in an *ad hoc* computer program where finger and thumb forces were printed to the computer screen at the end of each loop iteration.

### Grasping Task Performance

**Figure 3:**
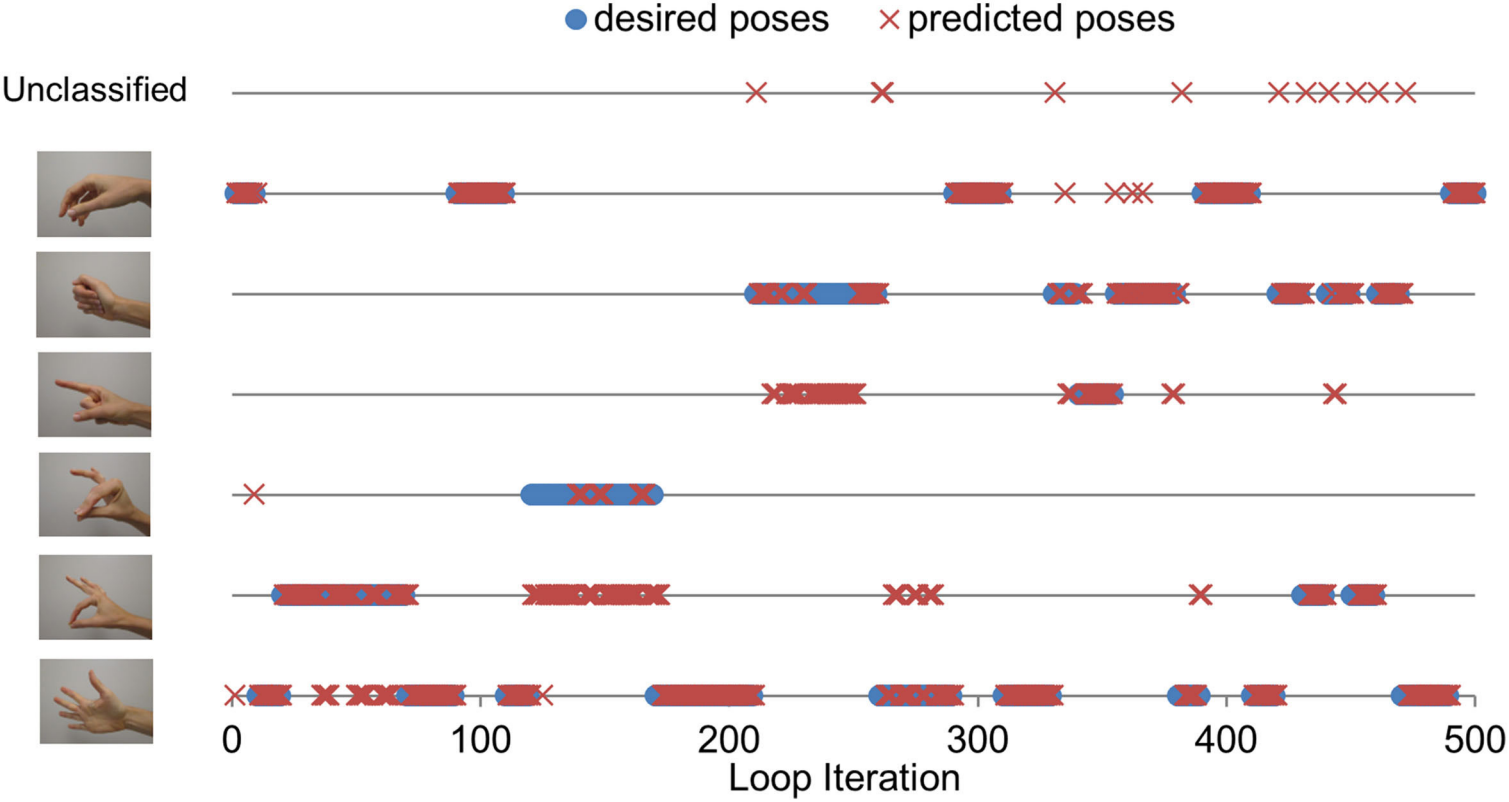
**Grasping task performance (third round, one subject)**. The figure shows the hand pose that the subject was instructed to achieve and maintain (blue circles), and the hand pose that the electromyographic (EMG) decoder predicted (red crosses). Each line corresponds to a specific hand pose. The top line refers to an unclassified hand pose, which happened when the EMG decoder output transitioned from one hand pose to the next.

Figure 3 illustrates task performance during the third round of virtual grasping tasks for a representative subject. Each of the five grasping task rounds consisted of 500 loop iterations, and each grasping task within a round consisted of 100 loop iterations (~300 ms/loop iteration). For the first 100 loop iterations, the subject was prompted to open the hand, do a 2-digit grasp, and then open the hand again. During the next 100 loop iterations, the subject was prompted to repeat with a 3-digit grasp. During loop iterations 200–300, the subject was prompted to perform a 5-digit grasp. During loop iterations 300–400, the subject was prompted to perform a 5-digit grasp, and then transition back and forth between a 5-digit grasp and an index finger point. During loop iterations 400–500, the subject was prompted to perform a 5-digit grasp, and then transition back and forth twice between a 5-digit grasp and a 2-digit grasp. The subjects were instructed to hold each hand pose for a fairly long duration to assess not only subjects’ ability to achieve a given hand pose but also to maintain it.

### Confusion Matrices

We created confusion matrices to illustrate the performance of the EMG decoder in predicting hand poses (Table 1). A confusion matrix not only indicates the overall accuracy of hand pose prediction but also specifies areas of confusion in predicting hand pose. Each entry of the confusion matrices is the median ± SEM across subjects. Entries in each row of the confusion matrix represents the hand pose that the subjects were instructed to adopt. Entries in each column represent the percentage of time that each hand pose was predicted. For example, the confusion matrix for Round 1 shows that when the subject was prompted to open the hand, an open hand was only predicted about 50% of the time (median across subjects). For 14% of the time, a 2-digit grasp was predicted instead. For 1% of the time, a 3-digit grasp was predicted. For 2 ± 6% of the time, a closed fist was predicted. An unclassified hand pose was predicted 5 ± 3% of the time.

**Table 1:** **Confusion matrices, rounds 1 to 5 ± interquartile range**.

Performed →	Open hand	2-digit grasp	3-digit grasp	Finger point	Closed fist	Rest	Unclassified
Desired ↓							
**Round 1: 63 ± 39% average success rate**							
Open hand	**50 ± 35**	14 ± 12	1 ± 1	0 ± 1	2 ± 22	0 ± 0	5 ± 9
2-digit grasp	6 ± 27	**72 ± 47**	4 ± 9	0 ± 0	4 ± 6	0 ± 0	3 ± 10
3-digit grasp	0 ± 2	11 ± 42	**48 ± 68**	0 ± 0	4 ± 8	0 ± 0	6 ± 24
Finger point	0 ± 0	0 ± 0	0 ± 0	**45 ± 37**	35 ± 37	0 ± 0	0 ± 13
Closed fist	0 ± 17	2 ± 3	0 ± 3	7 ± 15	**77 ± 26**	0 ± 2	5 ± 4
Rest	0 ± 0	3 ± 4	1 ± 1	1 ± 3	4 ± 7	**88 ± 21**	0 ± 4

**Round 2: 78 ± 36% average success rate**							
Open hand	**81 ± 19**	4 ± 7	1 ± 1	0 ± 1	2 ± 9	0 ± 0	1 ± 7
2-digit grasp	2 ± 19	**86 ± 56**	2 ± 3	0 ± 0	4 ± 6	0 ± 0	3 ± 9
3-digit grasp	0 ± 0	11 ± 36	**57 ± 62**	0 ± 0	2 ± 8	0 ± 0	4 ± 18
Finger pointing	0 ± 0	0 ± 0	0 ± 0	**49 ± 50**	31 ± 22	0 ± 0	0 ± 5
Closed fist	0 ± 2	0 ± 2	0 ± 1	0 ± 8	**93 ± 15**	0 ± 0	1 ± 43
Rest	0 ± 0	0 ± 0	0 ± 0	0 ± 0	0 ± 3	**100 ± 11**	0 ± 0

**Round 3: 82 ± 30% average success rate**							
Open hand	**78 ± 26**	3 ± 4	1 ± 1	0 ± 1	2 ± 6	0 ± 0	2 ± 3
2-digit grasp	3 ± 19	**85 ± 41**	1 ± 4	0 ± 0	4 ± 4	0 ± 0	3 ± 7
3-digit grasp	0 ± 2	7 ± 24	**74 ± 50**	0 ± 0	2 ± 4	0 ± 0	3 ± 16
Finger pointing	0 ± 0	0 ± 0	0 ± 0	**65 ± 45**	20 ± 7	0 ± 1	0 ± 13
Closed fist	0 ± 1	1 ± 2	0 ± 1	1 ± 3	**90 ± 8**	0 ± 1	2 ± 3
Rest	0 ± 0	0 ± 1	0 ± 0	0 ± 0	1 ± 3	**98 ± 11**	0 ± 0

**Round 4: 80 ± 21% average success rate**							
Open hand	**83 ± 28**	2 ± 1	1 ± 1	0 ± 1	2 ± 3	0 ± 0	2 ± 3
2-digit grasp	6 ± 11	**86 ± 34**	1 ± 1	0 ± 0	3 ± 4	0 ± 0	2 ± 1
3-digit grasp	0 ± 2	11 ± 24	**64 ± 32**	0 ± 0	1 ± 2	0 ± 0	5 ± 22
Finger pointing	0 ± 0	0 ± 0	0 ± 0	**57 ± 22**	27 ± 22	0 ± 0	0 ± 0
Closed fist	0 ± 1	1 ± 2	0 ± 0	1 ± 3	**93 ± 8**	0 ± 0	3 ± 3
Rest	0 ± 0	0 ± 0	0 ± 0	0 ± 0	1 ± 1	**99 ± 4**	0 ± 0

**Round 5: 83 ± 25% average success rate**							
Open hand	**86 ± 16**	4 ± 6	1 ± 1	0 ± 1	2 ± 3	0 ± 0	2 ± 2
2-digit grasp	6 ± 19	**83 ± 34**	2 ± 4	0 ± 0	4 ± 4	0 ± 0	3 ± 3
3-digit grasp	0 ± 0	7 ± 38	**73 ± 56**	0 ± 0	1 ± 8	0 ± 0	6 ± 16
Finger pointing	0 ± 0	0 ± 0	0 ± 0	**60 ± 22**	25 ± 20	0 ± 0	0 ± 0
Closed fist	0 ± 2	1 ± 2	0 ± 0	1 ± 3	**94 ± 9**	0 ± 0	1 ± 1
Rest	0 ± 0	0 ± 0	0 ± 0	0 ± 0	1 ± 1	**99 ± 16**	0 ± 0

*Numbers in bold represent the percent of the time that each hand pose was 5 correctly predicted*.

For an ideal discrimination of hand postures, a confusion matrix will have a value of 100% along the diagonal and a value of 0% off the diagonal. Non-zero percentages that are off of the diagonal represent areas of confusion between pairs of hand poses. Predictions of an unclassified hand pose occurred when transitioning from one classified hand pose to the next. Some unclassified poses included a closed fist with extension of the little finger, extension of the little finger and index finger, extension of the middle finger and ring finger, and flexion of only the thumb and middle finger. These poses occurred mostly when the subject was attempting an open hand, 2-digit grasp, or 3-digit grasp.

The confusion matrices for each round show an improvement in performance across rounds. However, it should be noted that for some subjects, the EMG decoder was retrained on one of the hand poses only after Round 1, thereby partially explaining the increase in performance from Round 1 to Round 2. From Round 2 to Round 5 however, there was some improvement in the ability to perform a 3-digit grasp and an index finger point that can be attributable only to practice.

Analysis of variance on the overall hand pose prediction accuracy revealed that prediction accuracy varied significantly across grasping tasks (*Time epoch*; *p* < 0.05) with no main effect of *Round* (practice) or interaction between *Round* and *Time epoch*. Although 5- and 2-digit grasping had the highest and lowest prediction accuracy, we found that no significant difference was found between these two grip types. However, we found a main significant effect of experimental session (*p* < 0.001).

### Achieving Target Forces

During each grasp, subjects were told to reach a target force. The total predicted grip force across the fingers in contact was summated for each loop/iteration. The subject was prompted to attain the predicted total grip force to match the target force as closely as possible. The task was challenging because of the large variation in predicted grip force from one loop iteration to the next. Although the predicted grip force was computed as the average of the previous three loop iterations, there still was significant variability in force predictions across consecutive loop iterations.

Quality of grip force control was measured by the NRMSE. Table 2 shows the NRMSE for each round of grasping tasks. The first row represents Round 1 and the bottom row represents Round 5 after subjects have undergone practice. The first column is the average NRMSE computed across all grasp types ± SEM. The other columns show the NRMSE for specific grasp types. The NRMSE shows little improvement with practice, but finer control of grip force during the 2-digit grasp. We found a marginally significant effect of number of digits when comparing force production with 2, 3, and 5 digits (*p* = 0.057), with NRMSE being lowest in the 2-digit case. This was most likely due to the fact that the 2-digit grasp only involves control of 2 of the digit flexion forces rather than all 5, leaving less room for variability in total grip force. The overall ANOVA revealed NRMSE from the 2-digit condition to be lower than 3- and 5-digit condition, whereas 3- and 5-digit conditions were not significantly different from each other. We found no interaction or effect of session on NRMSE.

**Table 2:** **Normalized root mean square error (NRMSE) of grip force control ± SEM**.

Experimental round	Average NRMSE (%)	NRMSE 2-digit (%)	NRMSE 3-digit (%)	NRMSE 5-digit (%)
Round 1	26 ± 5	24 ± 5	20 ± 3	26 ± 6
Round 2	23 ± 2	15 ± 2	26 ± 5	24 ± 2
Round 3	22 ± 2	18 ± 2	20 ± 2	23 ± 2
Round 4	22 ± 1	17 ± 2	23 ± 1	23 ± 2
Round 5	25 ± 3	18 ± 2	25 ± 3	25 ± 4

### Force Distribution across Fingers

**Figure 4:**
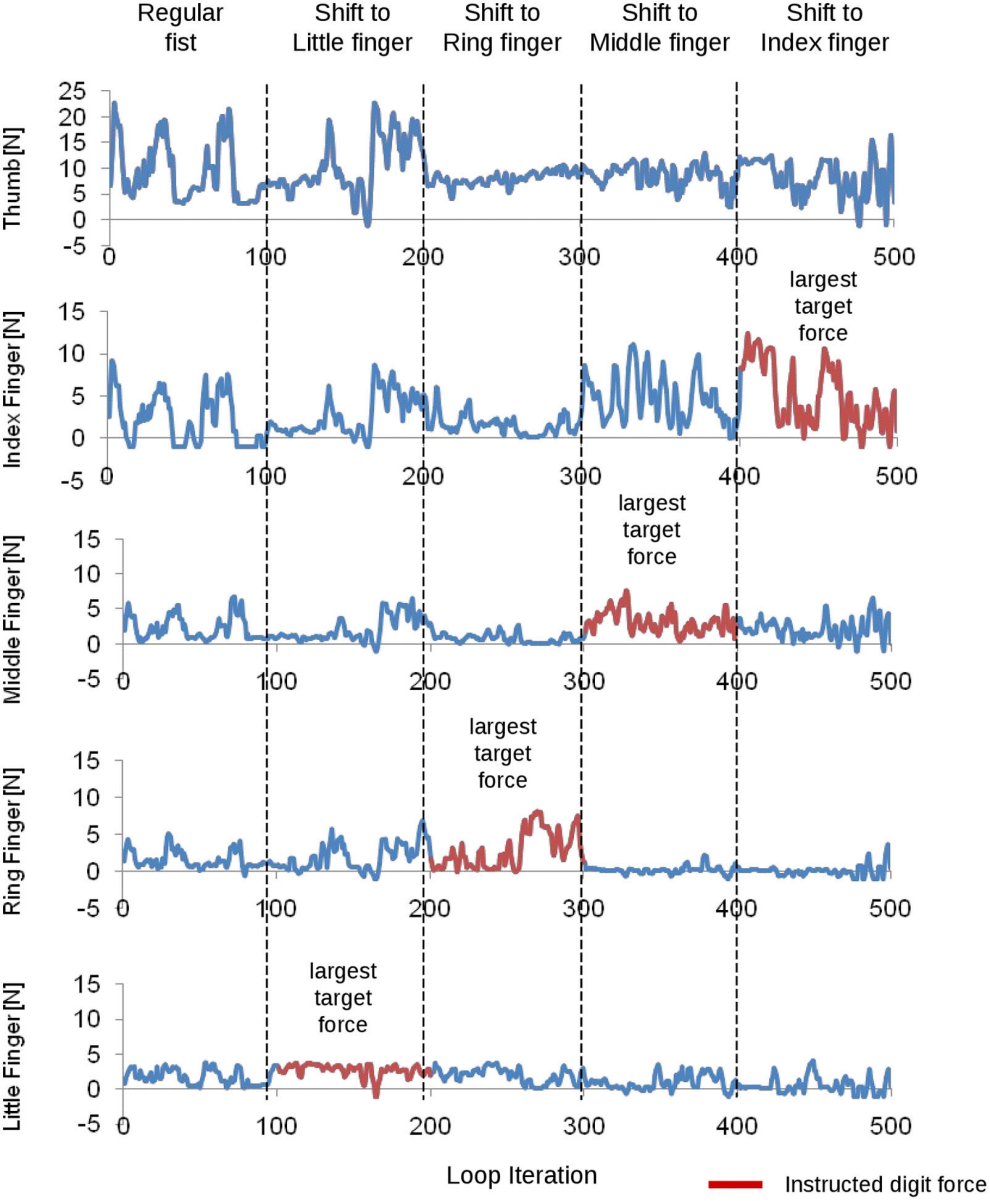
**Electromyographic decoder output for each finger flexion force (one subject)**. The subject was instructed to vary the force distribution across the fingers. The instruction given to the subject is shown on top for each time epoch. Time epochs are separated by vertical dashed lines. Data from each digit are shown on each row. The instructed digit for each task is denoted by a red trace. The little finger is expected to show an increased predicted force during loop iteration 100–200. During loop iteration 200–300, the ring finger is expected to show an increased predicted force. During loop iteration 300–400, the middle finger is expected to show an increased predicted force. During loop iteration 400–500, the index finger is expected to show an increased predicted force.

Figure 4 shows how the force distribution across the fingers changes across time epochs. For the first time epoch, subjects were told to make a fist and periodically vary the total force every 20 iterations. For the second time epoch, subjects were told to shift the grip force to the little finger. For the third time epoch, subjects were told to shift the force to the ring finger. For the fourth time epoch, subjects were told to shift the force to the middle finger. For the fifth time epoch, subjects were told to shift the force to the index finger.

**Figure 5:**
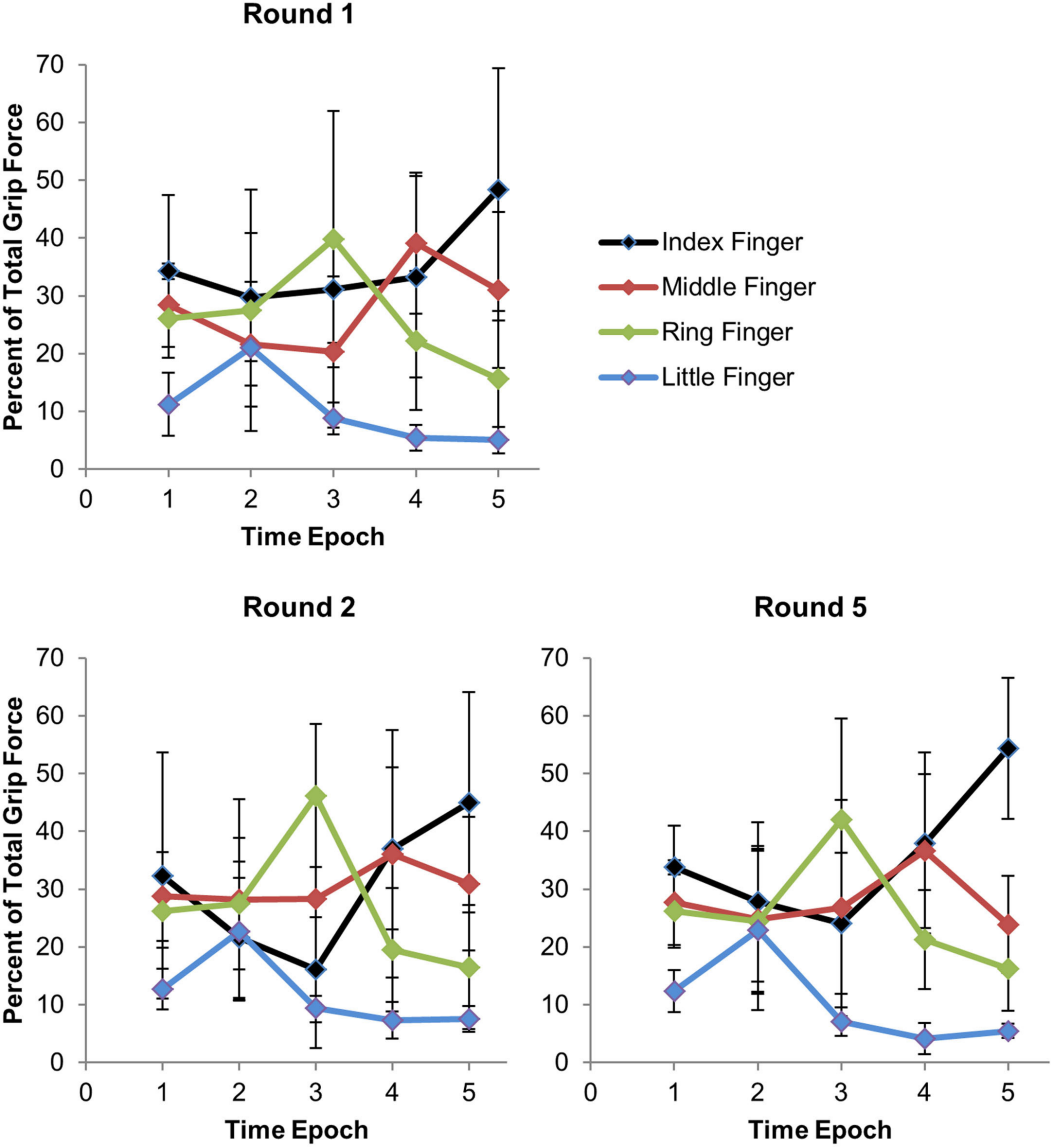
**Force distribution across fingers with each time epoch**. For epoch = 1, subject made regular closed fist. For epoch = 2, subject shifted force to the pinky. For epoch = 3, subject shifted the force to the ring finger. For epoch = 4, subject shifted the force to the middle finger. For epoch = 5, subject shifted the force to the index finger. Total grip force refers to the sum of the grip forces across the fingers, excluding the thumb. Error bars represent the interquartile range.

Figure 5 shows how the percent of total grip force on each finger varied across time epochs (horizontal axis). In time epoch 1, subjects were told to flex all of the fingers. Naturally, more force will be on the index finger and middle finger as these fingers can produce more force than other fingers. In time epoch 2, subjects were told to shift the force to the little finger. It can be seen that a shift in the force distribution toward the little finger occurred. In time epoch 3, subjects were told to shift the force to the ring finger. A substantial increase in predicted ring finger force is observed with a drop in predicted little finger force from time epoch 2. In time epoch 4, subjects were told to shift the force to the middle finger. A drop in predicted ring finger force is shown along with an increase in middle finger force and often an increase in index finger force. In time epoch 5, subjects were told to shift the force to the index finger. An increase in predicted index finger force is observed along with a decrease in middle finger force. The upper left plot represents data taken across subjects from Round 1. The lower left plot shows force distribution across time epochs in Round 2 after subjects have benefited from some practice and after retraining was done on one of the subjects for force predictions. The lower right plot represents data taken across subjects from Round 5 after subjects have benefited from more practice without any additional retraining. In Round 5, subjects are better at shifting more force selectively to the pinky and ring finger in time epochs 2 and 3. In both Rounds 1 and 5, subjects can easily shift force to the index finger and less on the little and ring finger.

Another way of illustrating grip force distribution across the fingers is the force distribution index, which is equal to −1 when all force across the fingers is concentrated on the pinky and equal to +1 when all force is concentrated on the index finger. When force is evenly distributed across the fingers, the force distribution index is 0. Force distribution index is computed as follows:

(9) $$F_{\text{dist}}=F_{\text{index}}*1+F_{\text{middle}}*0.5-F_{\text{ring}}*0.5-F_{\text{pinky}}*1$$

When the subject was told to make a fist during the first time epoch, we expected the force distribution index to be greater than 0 because naturally the index finger and middle finger each have a higher grip force than the little finger. Table 3 shows force distribution indices across time epochs (by row) and subjects (by column). Data from across rounds show more negative values when subjects were told to shift the force to the little finger, and greater positive values are found when subjects were told to shift the force to the index finger. Compared to when subjects were told to make a regular fist, the force distribution index was lower when subjects were told to shift the force to the ring or little finger, and higher when subjects were told to shift the force to the middle or index finger.

**Table 3:** **Force distribution indices for each subject and averages across subjects**.

	S1	S2	S3	S4	S5	S6	S7	S8	Average
**Round 1**
Regular fist	0.24	0.35	0.20	0.19	0.20	0.08	0.22	0.45	0.24
Shift to pinky	−0.10	0.32	−0.02	0.00	−0.22	0.54	−0.12	0.06	0.06
Shift to ring	0.10	−0.19	0.02	0.45	−0.26	0.73	0.14	0.01	0.12
Shift to middle	0.12	0.07	0.18	0.37	0.47	0.63	0.48	0.58	0.36
Shift to index	0.22	0.51	0.49	0.40	0.73	0.49	0.50	0.74	0.51
**Round 2**
Regular fist	−0.02	0.36	0.23	0.14	0.08	0.27	0.30	0.31	0.21
Shift to pinky	−0.13	0.40	0.10	0.13	−0.17	0.21	−0.42	−0.17	−0.01
Shift to ring	−0.02	−0.05	0.11	−0.10	−0.30	0.06	−0.04	0.15	−0.02
Shift to middle	0.39	0.20	0.17	0.19	0.61	0.52	0.38	0.58	0.38
Shift to index	0.38	0.49	0.27	0.23	0.56	0.31	0.60	0.73	0.45
**Round 5**
Regular fist	0.21	0.38	0.12	0.16	0.11	0.27	0.19	0.35	0.22
Shift to pinky	0.15	0.39	−0.00	0.20	−0.34	0.06	−0.31	0.25	0.05
Shift to ring	0.09	0.32	−0.07	0.38	−0.27	0.24	0.05	0.00	0.09
Shift to middle	0.62	0.14	0.06	0.51	0.62	0.40	0.46	0.51	0.42
Shift to index	0.32	0.54	0.54	0.42	0.66	0.42	0.70	0.61	0.53

We found that subjects were able to shift force distribution across the fingers from one time epoch to the next (significant main effect of *Time epoch*; *p* < 0.002). However, subjects did not improve with practice, and furthermore, we found that the duration of the time epoch did not affect how well subjects could maintain a given force distribution across fingers (no main effect of *Round, Sample*, or significant interaction; *p* > 0.1).

### Ability to Perform Grasping Tasks without Dropping the Object

A major factor influencing whether a grasping task can be performed successfully with an artificial hand is whether there is an unwanted opening of the hand as this can cause dropping of the object. In the trials involving grasping tasks, an opening of the hand when the hand was not supposed to be open rarely occurred (4.4 ± 1.4% of loop iterations), and there was little change in the incidence of unwanted hand openings across rounds. Unwanted hand opening occurred most often for the index-thumb grasp, as illustrated in the confusion matrices (Table 1). In trials involving shifts in force distribution across the fingers, unwanted hand opening also occurred very rarely (2.7 ± 1.2% of the time).

To further assess the ability to performing grasping tasks, subjects were allowed to myoelectrically control the i-limb hand. For these trials, subjects were instructed to do a few basic grasping tasks. The first task involved simply opening and closing the hand. The second and third tasks involved performing 2- and 3-digit grasps, respectively. The fourth task involved grasping with a 5-digit grasp, transitioning to index finger pointing, transitioning back to a 5-digit grasp, then transitioning to a 2- or 3-digit grasp before opening the hand. All subjects who attempted the opening and closing of the hand succeeded in performing these movements, although some subjects did so more easily than others. All subjects were able to do 2- and 3-digit grasps, but often with difficulty. All but one subject were able to perform the index finger pointing and transition from a 5- to a 3-digit grasp without losing contact. Subject 7 did not attempt the grasping tasks because of insufficient time availability.

Finally, we also asked subjects to report the level of difficulty they experienced while performing online control of each online i-limb task. 1 = “easy,” 2 = “able to do the task,” 3 = “able to do the task, but with effort,” 4 = “moderately hard,” and 5 = “very hard or could not do.” Table 4 shows the rating for each subject and task. More than half of the ratings (19/35) were below 3, i.e., subjects found the task to be easy or at least doable. Some of the ratings (9/35) were equal or greater than 3, indicating that subjects found the task moderately or very hard. These tasks were usually the finger pointing or the transition from 5- to 3-digit grasp. Although all subjects were able to transition from a 5- to a 3-digit grasp, some had trouble transitioning from a 5- to a 2-digit grasp without accidentally opening the hand. A 2-digit grasp involves flexing of 3 fingers, which could have caused the hand pose to be confused with the open hand pose, which involves flexing of all digits.

**Table 4:** **Subjective rating of difficulty level of i-limb grasping task**.

i-Limb tasks	S1	S2	S3	S4	S5	S6	S7	S8
5-digit grasp	2	2	1	2	2	1	n/a	1
2-digit grasp	3	2	3	2	1	1	n/a	1
3-digit grasp	4	3	2	2	1	1	n/a	4
Finger point	3	4	5	4	4	2	n/a	2
5→3 transition	5	3	4	3	3	1	n/a	3

## Discussion

This work has demonstrated a proof of principle for a system that decodes EMG signals from the upper limb of able-bodied subjects for online prediction of individual digit forces. Subjects were able to perform a variety of grasping tasks using an *ad hoc* computer program. The system is to eventually be used on transradial amputees for enabling them to perform grasping tasks and hand gesturing with myoelectric control of a prosthetic hand such as the i-limb hand.

Clearly, predicting individual digit forces alone is insufficient for online prediction of hand motion because the EMG-to-force mapping on a given digit changes depending on which of the other digits is flexed. For example, the EMG-to-force mapping for the middle finger is going to be different for a thumb-middle finger precision grasp versus a closed fist. By incorporating an SVM classifier that distinguishes between hand postures, myoelectric control of hand motion, and individual digit forces for everyday activities becomes more feasible.

As with any myoelectric control system, this system is vulnerable to changes in the EMG signal over time. This issue is particularly relevant for myoelectric control systems that predict multiple hand motions. As the proposed system has not been tested for its functionality over time periods of hours or days, future work will examine the sensitivity of our myoelectric control system to prolonged usage. Nevertheless, the impact of changes in EMG signals on myoelectric controllers has been extensively studied and the insight provided by this previous work could potentially be integrated with the proposed approach. Below we discuss previous work on myoelectric prosthesis controller algorithms and the contributions of the present work.

### Previous Work on Myoelectric Decoders

Previous work has also explored numerous techniques for using EMG signals to predict desired total grasp force and hand postures. With regard to grasp force prediction, Gijsberts and colleagues (16) demonstrated the use of a supervised non-linear incremental learning method (Incremental Ridge Regression) that makes occasional updates with small batches of training data each time. This approach led to a reduction in normalized mean square error and an increase in the correlation between desired and predicted grip forces.

With regard to hand kinematics, Anam and Al-Jumaily (17) used an online sequential learning method that used small chunks of finger movement data collected online as additional training data. These additional chunks of data were used to update the weights of the trained model without retraining the entire model. The online retraining allowed for a model prediction accuracy to be maintained at 85% day-to-day, whereas the system without online retraining could not. This system could distinguish among 10 movement classes, i.e., 5 individual finger movements and 5 combined finger movements where more than one finger flexed simultaneously. Another group (18) used supervised adaptation-based linear discriminant analysis methods to adapt to drifts in the EMG signal. Offline analysis showed improvements in classification accuracy from 75% without adaptation to 92% with adaptation. For online control, the accuracy increased by 25%. The system distinguished between a mix of hand and wrist movements, which included wrist pronation/supination, wrist flexion/extension, hand open, 2-digit pinch grip, key grip, and no movement. Al-Timemy and colleagues (19) demonstrated classification of up at 15 finger movements with 98% accuracy using only 6 EMG channels in intact subjects and 90% accuracy using 11 EMG electrodes in amputees. Interestingly, the system showed better performance using Orthogonal Fuzzy Neighborhood Discriminant Analysis (OFNDA) for feature selection than principal component analysis (PCA). Unlike PCA, OFNDA takes into account maximum separation of feature values between classes. PCA only takes into consideration maximum variability among feature values in feature space.

Continuous morphing between hand postures has been another attempt at making prosthetic hand movements more natural. Segil and Weir (20) created a mapping between the EMG principal component and joint angle domains for allowing real-time control by able-bodied subjects of 15 joints of a virtual hand displayed on a computer screen. The authors reported accurate control of 13 out of 15 joints in the best-case scenario. In addition, EMG control and joystick control were found to be comparable in controlling joint motions. By using SVMs, Khushaba et al. (21) distinguished 10 classes of individual and combined finger movements, including a hand close movement, with 90.3% accuracy in real-time control experiments on eight able-bodied subjects.

Finally, ultrasound imaging has also been studied as a means to overcome the limitations of surface EMG. Akhlaghi and colleagues (22) implemented a real-time control system that classifies hand motions by sensing mechanical deformations in forearm muscle compartments. Although the results were encouraging, the system is not feasible for eventual integration with hand prosthesis controllers.

### Contributions to Previous Work

It should be noted that the above-cited work on myoelectric algorithms has focused mostly on EMG-based prediction of total grasping force. To the best of our knowledge, the use of myoelectric signals to predict individual finger forces has not been investigated. This is a major gap, as successful prediction of flexion force of *individual* fingers from EMG signals could be useful for performing in-hand object manipulation using a prosthetic hand. Another gap in the literature on myoelectric controller algorithms is that there has been no demonstration of smooth transitions between grasp types without losing contact with the object. Although previous work has explored myoelectric control of smooth transitions between hand postures (20), such work did not address how easily such transitions between grasp types could be made without accidental loss of contact with the object.

In the present work, we have demonstrated proof of principle of a unique system that provides online intuitive control of individual finger forces. Subjects simply press their fingers into the palm of their hands, and the force exerted by each finger into the palm is approximately correlated with the flexing force command delivered to the corresponding finger of the robot hand. The training process involves grasping a force-sensing object only with the contralateral hand while pressing with approximately identical finger flexion forces on the ipsilateral hand. In this way, the system could be trained on amputees who have no ipsilateral hand with which to grasp the force-sensing object.

Although the RFR that predicted each finger force was trained only when the subject was making a fist, finger force prediction was transferable to other grasp types such as the 2-digit pinch grip. As long as the subject was given feedback of the digit forces, the subject was able to modulate his/her EMG signals to adjust the total grasping force accordingly. For grasping tasks performed using a computer program to display predicted finger forces, confusion matrices show that most of the improvement in performance across rounds was from Round 1 to Round 2, with smaller improvements shown in subsequent rounds (Table 1). In Round 5, our algorithm attained success rates ranging from 60% (index finger pointing) to 94 and 99% for closed fist and resting state, respectively. Some of the improvement from Round 1 to Round 2 was due to retraining on 1–2 hand poses on some of the subjects. Some improvement could thus be attributed to practice and familiarization to the task. Overall, our results show that subjects have voluntary control over the hand pose that the system outputs, although there is room for improvement in the system’s prediction accuracy.

Figures 4 and 5 demonstrate the feasibility of EMG control of not only total grip force but also grip force distribution across the fingers during a grasping task. By selectively pressing the fingers into the palm of the hand, subjects can control which fingers have the largest normal force at the system output. This feature of the system allows for more dexterous control of individual finger motions that is intuitive for the user. However, due to high noise in predictions of total grip force, NRMSE is higher than it could have been. Finally, we demonstrate that subjects can use myoelectric control to transition between grasp types without losing contact with the object, as shown in Figure 3.

### Methodological Considerations

One way of improving the system is incorporation of a Kalman filter for hand posture predictions. A Kalman filter can take into account the degree of uncertainty in each hand posture prediction, and it gives higher weight to predictions with higher levels of certainty. A one-against-one SVM selects the hand posture with the most votes. Therefore, the first possible measure of uncertainty in the SVM is the number of votes in favor of the chosen hand posture relative to the number of votes in favor of the other hand postures. If there are two hand postures that both have the largest number of votes, then there is uncertainty in the hand pose prediction. The second possible measure of uncertainty is in the distance of a data point from the dividing hyperplane in feature space. For distinguishing each pair of hand poses in an SVM, there is a dividing hyperplane in feature space that assigns a specific hand posture to data points on one side of the hyperplane and another hand posture to data points on the other side of the hyperplane. The shorter the distance between a data point and the dividing hyperplane, the larger the degree of uncertainty, and therefore the lower the weight that would be given to that prediction. A third measure of uncertainty would be whether the prediction of hand posture is different from the previous few predictions. When the system has a high enough processing speed to make 20 predictions/second, each prediction can be weighed differently depending on its level of uncertainty.

Even though the trials performed using a computer program to display predicted finger forces were characterized by an unwanted open hand in a small percentage of the trial duration, when using the i-limb it only takes one unwanted hand opening of the hand to classify the grasping task as unsuccessful. As described above, there are several methods that can be implemented for preventing unwanted opening of a prosthetic hand during a real grasping task. One such method is a Kalman filter, which weighs different hand pose predictions depending on their level of uncertainty.

With regard to our delay in hand posture estimation, Farrell and Weir (23) estimated 100–125 ms as optimal delays for fast and slower prehensors, respectively. The optimality of these delays is based on compromising between allowing for sufficient time for EMG decoding and maximizing the responsiveness of the prosthesis. Although our 300-ms delay is more than twice the optimal delay identified by Farrell and Weir (23), it could be significantly improved by at least 10-fold by using a different software platform (i.e., C++ instead of Matlab) to enable a larger number of hand pose predictions to be made per second. Using Kalman filters (see above) would allow for the weighing of different hand pose predictions based on the level of certainty in each prediction.

### Potential Applications for Individuals with Bilateral Upper Limb Loss

Some individuals may have both hands amputated, in which case they have no hand with which to grasp a force-sensing device. In such cases, the system can make assumptions about the finger flexion forces at certain time points. For example, when the amputee is asked to concentrate the force on a specific finger, the system can assume an arbitrarily higher flexion force for that finger and a minimal force on the other fingers during that time frame. Amputees also can be prompted to vary the total grip force of a grasp where in some time frames the amputee can be prompted to exert a minimal total grasp force, and in other time frames the amputee can be prompted to exert a high grasping force. For each time frame, the system can make an assumption about the total grasp force and assume how the force would be distributed across the fingers during a grasp such as a regular closed fist depending on previous data from able-bodied individuals. For each case, the opposing thumb force can be assumed to be approximately equal to the summation of the four finger forces. An advantage of this approach is that it requires the same effort across subjects for exerting specific grasping forces because the assumption made of the grasping force for each time frame is independent of the magnitude of EMG signal (because of normalization) and of the overall muscular strength of the subject.

## Conclusion

We have demonstrated proof of principle in the use of five EMG electrodes for predicting hand pose and individual finger forces using a one-against-one SVM and RFR, respectively. The present system has potential for myoelectric control of dexterous hand prostheses. Future work should explore additional methods of feature selection, signal filtering, machine learning classification, Kalman filtering, and training. New myoelectric control systems should be adjustable with small amounts of new training data without the need to retrain the entire system so that drifts in the EMG signal over time do not decrease classification accuracy.

## Ethics Statement

Subjects gave informed written consent to participate in the experiments. The experiments were approved by the Institutional. Review Board at Arizona State University (Protocol: #1201007252) and were in accordance with the Declaration of Helsinki. Prior the experiment, participants were given time to read a consent form that described the experimental procedure. After reading the form, participants were asked if they had any questions. After any questions were answered, participants were asked to sign and date the consent form. Next, the experimenter signed and dated the consent form. No participants from vulnerable populations were used in this study.

## Author Contributions

AG designed the prosthetic control system, designed most of the experimental protocol, carried out the experimental protocol, collected data, analyzed data, and made data plots. PA provided expertise in the area of myoelectric control of robotic devices, thereby playing a role in design of the prosthetic control system and experimental protocol design. MS provided expertise in motor control and neuroscience. He contributed to the experimental design and data analysis methods. All authors contributed to manuscript preparation.

## Discalimer

The content is solely the responsibility of the authors and does not necessarily represent the official views of the National Institutes of Health.

## Conflict of Interest Statement

The authors declare that the research was conducted in the absence of any commercial or financial relationships that could be construed as a potential conflict of interest. The reviewer AA and handling editor declared their shared affiliation and the handling editor states that the process nevertheless met the standards of a fair and objective review.
